# Retinopathy of Prematurity in Eight Portuguese Neonatal Intensive Care Units: Incidence, Risk Factors, and Progression—A Prospective Multicenter Study

**DOI:** 10.3390/children11101154

**Published:** 2024-09-24

**Authors:** Mariza Fevereiro-Martins, Ana Carolina Santos, Carlos Marques-Neves, Manuel Bicho, Hercília Guimarães, on behalf of the GenE-ROP Study Group

**Affiliations:** 1 Ecogenetics and Human Health Unit, Environmental Health Institute-ISAMB, Associate Laboratory TERRA, Faculty of Medicine, University of Lisbon, Av. Professor Egas Moniz, 1649-028 Lisboa, Portugal; 2 Institute for Scientific Research Bento da Rocha Cabral, Calçada Bento da Rocha Cabral 14, 1250-012 Lisboa, Portugal; 3 Department of Ophthalmology, Cuf Descobertas Hospital, Rua Mário Botas, 1998-018 Lisboa, Portugal; 4 Center for the Study of Vision Sciences, University Ophthalmology Clinic, Faculty of Medicine, University of Lisbon, Av. Professor Egas Moniz, Piso 1C, 1649-028 Lisboa, Portugal; 5 Department of Gynecology—Obstetrics and Pediatrics, Faculty of Medicine, University of Porto, Alameda Prof. Hernâni Monteiro, 4200-319 Porto, Portugal

**Keywords:** retinopathy of prematurity, preterm infant, premature birth, risk factor, red blood cell transfusions, screening

## Abstract

Background/Objectives: Retinopathy of prematurity (ROP) is a retinal neovascular disease affecting preterm infants. Identifying risk factors for its development and progression is critical for effective screening and prevention. This study aimed to analyze the incidence of ROP and identify key risk factors for its development and progression. Methods: We conducted a prospective, observational cohort study on 455 neonates (gestational age [GA] < 32 weeks or birth weight < 1500 g) across eight Portuguese NICUs. Results: ROP incidence was 37.8%, with 4.6% requiring treatment. Multivariate analysis identified low GA and the number of red blood cell (RBC) transfusions as significant factors for ROP development and progression. After adjusting for these variables, platelet transfusions, high maximum fraction of inspired oxygen (FiO_2_) in the second week, and surfactant use remained significantly associated with ROP development, while early and late sepsis, maternal chronic hypertension, and delayed enteral nutrition were associated with progression to ROP requiring treatment. Conclusions: These findings underscore the importance of addressing low GAs and adult RBC transfusions in ROP risk management and suggest that maximum FiO_2_, platelet transfusions, and sepsis also play crucial roles. Larger studies are needed to validate these results and explore preventive interventions, particularly regarding the impact of multiple adult RBC transfusions on fetal hemoglobin percentages.

## 1. Introduction

Retinopathy of prematurity (ROP) is a retinal neovascular disorder primarily affecting preterm infants due to the immaturity of their retinal vasculature [1]. While advances in neonatal care, fueled by scientific and technological innovations, have markedly increased the survival rates of infants born at lower gestational ages (GAs), they have also contributed to a higher incidence of ROP [2]. Notably, ROP is recognized as the second leading cause of childhood blindness, surpassed only by cortical visual impairment, as documented in studies [3].

ROP progresses through two phases: Initially, retinal vessel growth is arrested due to postnatal hyperoxia [4]. Subsequently, the incompletely vascularized retina becomes hypoxic, leading to neovascularization driven by angiogenic growth factors [5]. 

The etiology of ROP is multifactorial, with a low GA and hyperoxia identified as primary contributors [6]. Additionally, genetic factors have been implicated in the development of the disease, as evidenced by studies estimating the heritability of ROP in twins and identifying polymorphisms in genes such as the *brain-derived neurotrophic factor* (*BDNF*) associated with severe ROP [7,8]. Our previous research further suggests that genetic factors may influence susceptibility to ROP by regulating erythropoiesis and fetal hemoglobin (HbF) expression, potentially through polymorphisms in genes involved in epigenetic regulation, including *lysine-specific histone demethylase 1A* (*KDM1A*) [9].

ROP is categorized based on the International Classification of Retinopathy of Prematurity (ICROP), most recently updated in 2021 with the ICROP3 revision [10,11,12]. The ICROP provides a comprehensive system for describing the acute changes in ROP based on four key parameters: the location of retinal involvement (zones), the presence of plus disease (indicating vascular dilation and tortuosity), severity (stages), and the extent of disease (measured in clock hours). The retinal area is organized into three concentric zones centered around the optic nerve: Zone I, the innermost and most crucial region, extends to a radius that is double the distance between the optic nerve and the macula; Zone II extends from the edge of Zone I to the ora serrata nasally and an equivalent distance temporally; and Zone III, the outermost zone, forms a crescent-shaped peripheral area. Plus disease, characterized by increased vascular dilation and tortuosity, can occur in any zone, with the highest risk of poor outcomes in Zone I and significantly lower risk in Zone III [13].

ROP progresses through five stages, beginning with mild vessel growth and a demarcation line in stage 1, progressing to a ridge in stage 2, and involving neovascular tissue in stage 3, where abnormal vessels extend into the vitreous. Stage 4 features partial retinal detachment, and stage 5 is characterized by total retinal detachment, potentially leading to blindness [14]. For treatment purposes, ROP is further categorized into two types: Type 1 ROP, which is more severe and requires prompt treatment, includes ROP in Zone I with plus disease, stage 3 in Zone I without plus disease, and stage 2 or 3 in Zone II accompanied by plus disease. Type 2 ROP is less severe and can often be managed with observation; it includes stage 1 or 2 ROP in Zone I without plus disease, and Zone II, stage 3 ROP without plus disease [12]. The main treatments for ROP—laser photocoagulation and intravitreal injections of anti-vascular endothelial growth factor (anti-VEGF) agents—focus on the proliferative phase of the disease, which leads to neovascularization rather than the initial phase [15,16]. Preventive measures are essential given the risks associated with treatment and the potential for long-term functional consequences [15,17,18].

Current screening guidelines primarily rely on birth weight (BW) and GA. However, numerous studies have proposed additional risk factors for ROP development [19]. Notably, few studies have differentiated between risk factors for the onset of ROP and those associated with its progression—an understanding critical for developing effective preventive strategies.

This study aims to analyze the incidence of ROP, as well as the risk factors contributing to both the development and progression of the disease.

## 2. Materials and Methods

### 2.1. Ethical Approval

The Scientific Council of the Faculty of Medicine of the University of Lisbon, along with the scientific councils of all participating hospital centers, the Ethics Committee of Centro Hospitalar Universitário de Lisboa Norte and Centro Académico de Medicina de Lisboa (CAML), as well as the ethics committees of all participating hospital centers, have granted their approval for this study. Written informed consent was obtained from parents. The study complied with the tenets of the Declarations of Helsinki and Tokyo.

### 2.2. Study Design and Population

This prospective, multicenter, and observational cohort study (ISRCTN16889608) included preterm infants born in eight Neonatal Intensive Care Units (NICUs) at hospital centers in the north, center, and south of Portugal. The study was conducted in phases in the different hospital centers between 19 November 2018 and 21 July 2021.

The inclusion and exclusion criteria were previously explained [20]. All infants born with a GA < 32 weeks or a BW < 1500 g were included. Infants with ocular diseases other than ROP (except conjunctivitis, keratitis, and congenital nasolacrimal duct obstruction), those with no known outcome of ROP, or those with insufficient clinical data were excluded.

### 2.3. Data Collection

Demographic, laboratory, and clinical data were collected. Biochemical parameters during the first week of life were determined using standardized methods.

Neonatal data included the following:

(1) Birth data: mode of delivery, GA, gender, body weight, small for GA (SGA) (BW percentile < 10), Apgar score, and need for resuscitation with endotracheal intubation.

(2) Postnatal data included the following:

- Pathologies, such as early sepsis (onset in the first 72 h of life), late sepsis (onset after the first 72 h of life), moderate and severe bronchopulmonary dysplasia [21], peri-intraventricular hemorrhage grade ≥ 2 [22], cystic periventricular leukomalacia [22], hemodynamically significant patent ductus arteriosus [23], necrotizing enterocolitis, and phototherapy.
- Treatments, including days of mechanical ventilation (invasive and non-invasive) and maximum fraction of inspired oxygen (FiO_2_), surfactant use, erythropoietin or darbepoetin, systemic and inhaled corticosteroid, non-steroidal anti-inflammatory, diuretic, red blood cell (RBC), platelets, and plasma transfusions received.
- Metabolic acidosis (considered if pH < 7.2 and bicarbonate < 16 mmol/L) measured on the first day of life at two time points (within the first 2 h and between 2 and 24 h).
- Body weight throughout the first month of life.
- Number of days with hyperglycemia (blood glucose > 125 mg/dL, measured every 8 h using a point-of-care glucometer) in the first three weeks of life.
- Nutritional data, including the day of the start of trophic, nutritious and total enteral nutrition, as well as breast milk feeding.
- Length of hospital stay (in days).
- Biochemical parameters during the first week of life: serum urea (mg/dL), creatinine (mg/dL), and total and direct bilirubin (mg/dL).

Maternal data included maternal age, family ancestry, level of education, behavioral habits, obstetric history (previous pregnancies, use of assisted reproductive technologies, single or multiple gestations), and maternal pathologies such as chronic or pregnancy-induced hypertension (gestational hypertension with or without preeclampsia/eclampsia), diabetes, and chorioamnionitis.

### 2.4. ROP Screening and Ophthalmological Data Collection

ROP screening and ophthalmological data collection were previously described [20]. An initial retinal examination was performed using indirect ophthalmoscopy or digital fundus retinography at 32 weeks of postmenstrual age or between 4 and 6 weeks of life, whichever occurred later. Scheduled follow-up examinations were conducted for each case based on the presence, severity, and location of ROP. These examinations were repeated until complete retinal vascularization or remission of ROP. Initial and follow-up assessments were recorded following the International Classification of ROP Revisited [11]. The indication for treatment of ROP followed the Early Treatment for ROP Study [24]. All infants diagnosed with type 1 ROP were treated with laser photocoagulation or anti-VEGF. The choice of treatment depended on the location and stage of the disease, the general condition of the infant, and the hospital center.

### 2.5. Statistical Analysis

Descriptive data for each categorical variable were presented as the absolute frequency and the corresponding proportion. The Pearson χ2 test was used to evaluate the significant differences between groups. The median and interquartile range or mean and standard deviation were presented for continuous variables. If the variables had normal distribution and homogeneity, the Student’s *t*-test was used; if not, Mann–Whitney was used to compare groups. To assess the risk factors for ROP versus No ROP or ROP not requiring treatment versus Type 1 ROP, a logistic regression analysis and the corresponding 95% CI were calculated. We first performed univariate analysis to approach relations between independent variables (GA, BW, Apgar score 5th minute, number of days with hyperglycemia, days of oxygen supplementation, RBC and platelets transfusions, bronchopulmonary dysplasia, persistent ductus arteriosus, sepsis, necrotizing enterocolitis, preeclampsia/eclampsia, and gestational diabetes) and outcomes (ROP vs. No ROP or ROP not requiring treatment vs. Type 1 ROP). When univariate analyses yielded a *p*-value < 0.1, those variables were incorporated into multivariable logistic regression models. Statistical analysis was executed with the IBM^®^ SPSS^®^ Statistics version 28.0 for Windows^®^, with a significant value of *p* < 0.05.

## 3. Results

### 3.1. Baseline Clinical Information

During the recruitment period, 647 infants met the inclusion criteria. Not including 42 infants without informed consent and 150 due to reasons that precluded complete data collection (62 deaths, 54 hospital transfers, 33 incomplete records, and 1 enrolled in another study), 455 infants were included in the final cohort. The median GA was 29.6 ± 3.2 weeks, and the median BW was 1175.7 ± 445.0 g, with 50.5% (*n* = 230) female (Table 1). Additional detailed data can be found in the supplementary materials (Supplementary Table S1).

### 3.2. Factors That Were Influencing ROP Development and Progression

**Figure 1:**
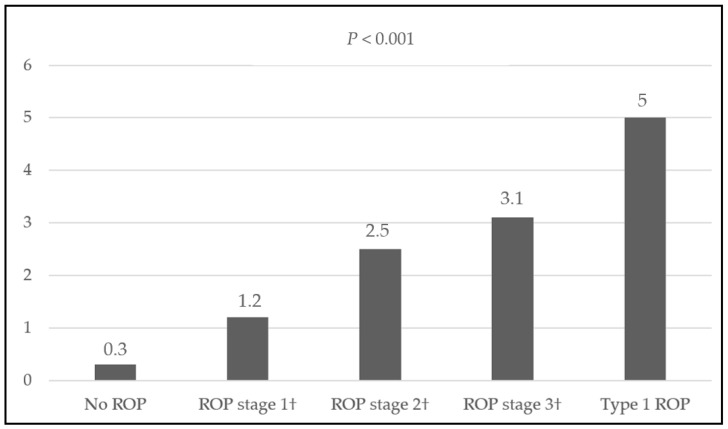
Stage and severity of ROP in function of the mean number of RBC transfusions. † Patients who do not meet the criteria for Type 1 ROP. *P*, Chi-square test.

Table 1 and Table 2 show the cohort’s demographic and clinical characteristics and their correlation with the development of ROP and its progression to ROP requiring treatment (type 1 ROP). Additional detailed data for Table 2 can be found in the supplementary materials (Supplementary Table S2). Table 3 shows maternal demographic and prenatal characteristics and their association with ROP development and progression. Further details are available in Supplementary Table S3. An increased mean number of RBC transfusions was significantly associated with a higher severity of ROP (*p* < 0.001). As depicted in Figure 1, infants with more severe ROP, including type 1, required more transfusions compared to those with milder stages or no ROP. The mean number of RBC transfusions progressively decreased from type 1 to stages 3, 2, and 1, with the lowest in infants without ROP. Logistic regression identified GA and the number of RBC transfusions as significant risk factors for both the development and progression of ROP. The clinical and demographic characteristics shown in Table 1, Table 2 and Table 3 were adjusted for these risk factors.

Therefore, GA, the number of RBC and platelet transfusions, maximum FiO_2_ at birth and in the second week of life, surfactant administration, and duration of hospitalization were significantly and independently associated with the development of ROP. Unexpectedly, being SGA was independently associated with a reduced likelihood of ROP development.

The variables significantly and independently associated with progression to ROP requiring treatment were GA, number of RBC transfusions, dystocic birth, resuscitation with intubation, early and late sepsis, day of initiation of trophic and nutritive enteral nutrition, Portuguese ancestry, level of mother education, assisted reproduction techniques, and maternal chronic arterial hypertension.

### 3.3. Characteristics of ROP

The incidence of ROP was 37.8% (172/455), and 4.6% (21/455) of the infants developed type 1 ROP. ROP stages 1 and 2 were the first and second most frequent, respectively (Table 4). None of the infants had ROP stage 4 or 5.

**Figure 2:**
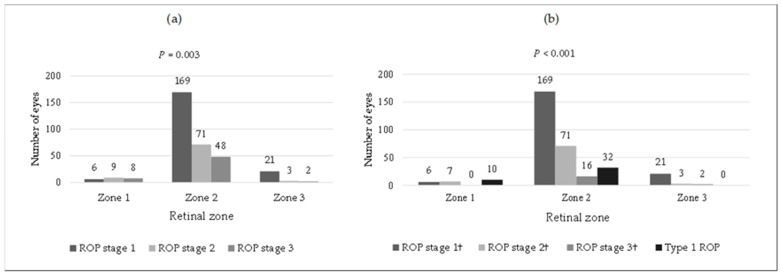
Retinal zone distribution of the different stages of ROP: (**a**) regardless of the need for treatment; (**b**) considering the need for treatment (type 1 ROP). † Patients who do not meet the criteria for Type 1 ROP. *P*, Chi-square test.

The mean postmenstrual age at the time of ROP diagnosis was 33.4 weeks in the group that developed type 1 ROP, slightly earlier than in those who did not require treatment for ROP, where the mean was 34.2 weeks. The postmenstrual age at the maximum stage of ROP was higher in infants who developed type 1 ROP, averaging 36.5 weeks, compared to 35.3 weeks in those who did not develop type 1 ROP. There was a statistically significant difference in the distribution of ROP across zones, with Zone II showing the highest prevalence (85.5%) (Figure 2). Additionally, the stages of ROP differed significantly across the zones (*p* = 0.003), as did the comparison between type 1 ROP and other stages (*p* < 0.001). In Zone I, only 6 infants were in stage 1 ROP, while 9 and 8 infants were in stages 2 and 3, respectively. Zone II was the most prevalent, encompassing all stages of ROP, whereas Zone III included only cases of stage 1 ROP. Type 1 ROP was identified in 42 eyes, with 76.2% occurring in Zone II and none in Zone III. Nearly all eyes with type 1 ROP were at stage 3, with only two at stage 2 (Table 4). Fourteen eyes were treated with anti-VEGF (bevacizumab), twenty-four with laser photocoagulation, and four initially received anti-VEGF but later required laser photocoagulation. Among these four, one subsequently required surgery due to vitreous hemorrhage. Anti-VEGF was administered for all cases of Type 1 ROP in Zone I and posterior Zone II. Importantly, no infant with stage 1 ROP required treatment.

## 4. Discussion

The incidence and severity of ROP vary significantly across countries, influenced by the quality of neonatal care and the availability of effective screening and treatment programs [25]. Comparing ROP incidence in population-based studies is challenging due to differences in study methodologies, survival rates, infants’ GA, and treatment protocols [1].

A recent meta-analysis reported an overall ROP prevalence of 31.9%, with 7.5% classified as severe ROP [26]. While overall ROP rates have remained stable over the past four decades (1985–2021), preterm infants with a GA of 28 weeks or less showed higher incidence. Lower-middle-income countries had higher overall ROP prevalence, whereas high-income countries had more severe cases [26].

In our study, 37.8% (172/455) of infants developed ROP, with 4.6% (21/455) requiring treatment. This study represents the most extensive prospective study on ROP in Portugal, encompassing eight NICUs.

A previous single-center prospective study in Portugal found that 21.7% (33/152) of neonates developed ROP, with 3.9% (6/152) requiring treatment [27]. Most Portuguese ROP studies are single-center and retrospective, resulting in varying incidence rates [28,29,30].

Multivariate regression analysis identified GA at birth and the number of RBC transfusions as crucial risk factors for ROP development and progression, highlighting their predominant role in ROP pathogenesis compared to other known risk factors. Notably, infants with a GA ≤ 28 weeks accounted for 65.1% of all ROP cases and 100% of those requiring treatment, consistent with findings from other studies that emphasize GA as a critical determinant of both the occurrence and severity of ROP [31]. This is likely due to the incomplete retinal vascular development in extremely preterm infants, which increases their vulnerability to oxidative stress.

Several studies have demonstrated a strong association between RBC transfusions and ROP [32]. Transfusing adult RBCs reduces the HbF to adult hemoglobin A (HbA) ratio, with this effect becoming more pronounced as the number of transfusions increases [32]. This reduction in HbF shifts the oxygen dissociation curve to the right, enhancing oxygen delivery to the retina, leading to increased oxidative stress and a reduced angiogenic stimulus [33]. HbF is critical in mitigating oxidative stress by preventing heme release and demonstrating superior pseudo-peroxidase activity, rapidly converting reactive ferryl-heme compared to HbA [34]. Our previous research suggested that a polymorphism in the *lysine-specific histone demethylase 1A* (*KDM1A*) gene, which regulates HbF suppression and erythroid maturation, might influence ROP susceptibility by affecting erythropoiesis and the transition from HbF to HbA [9]. Interventions that slow the decline in HbF following adult RBC transfusions should be explored to reduce ROP incidence and severity [34]. Approaches such as delayed umbilical cord clamping, blood-sparing sampling techniques, and the use of umbilical cord blood for transfusions show promise in maintaining physiological HbF percentages, potentially mitigating ROP risk [32,35,36]. The potential benefit of utilizing umbilical cord blood for transfusions is currently being investigated in a multicenter randomized trial (NCT05100212) [37].

While some variables, such as the frequency of RBC transfusions and GA, may represent secondary associations, the robust sample size provides strong statistical power to confirm their relevance. Our findings highlight the critical role of RBC transfusions and a low GA in ROP development and progression. The number of platelet transfusions was significantly associated with the development and progression of ROP, but only independently with its development. Several studies have linked platelet transfusions to ROP [38]. Similar to anemia, thrombocytopenia in preterm infants may result from a marked deficiency in erythropoietin production due to their immature kidneys, with the severity increasing as the GA at birth decreases [39]. Erythropoietin is an oxygen-regulated growth factor that, in addition to stimulating erythropoiesis and promoting angiogenesis, has anti-apoptotic effects and plays a role in thrombopoiesis [40,41]. Additionally, platelets contain crucial angiogenic factors; therefore, thrombocytopenia can impair angiogenesis in the incompletely vascularized retina [42].

Low BW is a recognized risk factor for ROP, although its impact varies across studies [43]. Our study confirmed a significant association between lower BW and both the development and progression of ROP, with this relationship being influenced by GA at birth and the number of RBC transfusions.

SGA was more prevalent among infants who did not develop ROP than among those who did. This finding may be attributed to the study’s criteria, which included SGA infants with a higher GA, allowing for more advanced retinal vascular development despite their lower BW. In contrast, a study in Brazil found no significant association between SGA and ROP [44].

Since ROP was first described, its development has been strongly linked to oxygen exposure [45]. Numerous studies have correlated ROP with high FiO_2_ levels and prolonged mechanical ventilation [46]. Our study further supports these findings, showing that higher maximum FiO_2_ levels during the first three weeks of life and extended mechanical ventilation significantly influence ROP development. Interestingly, the association between ROP and maximum FiO_2_ was independent at birth and in the second week of life. However, this association was not independent in the first week, likely due to the complex interplay of factors affecting ROP during this early period.

We identified a significant and independent association between surfactant use and ROP development, likely due to the increased need for supplemental oxygen in infants treated for respiratory distress syndrome (RDS) [47,48].

Our study highlights the significance of both early and late sepsis as independent risk factors for the progression of ROP. Systemic inflammation may predispose infants to ROP, with several studies identifying it as a crucial risk factor [49,50]. Sepsis can trigger the production of cytokines, chemokines, and growth factors, impairing angiogenesis and worsening retinal ischemia [51]. This process is often accompanied by hypotension, further compromising retinal perfusion [52]. In animal models of ROP, systemic inflammation has also been shown to disrupt retinal blood vessel development, leading to abnormal vascularization [53].

Delaying enteral nutrition was associated with ROP progression, suggesting the potential benefits of early feeding for at-risk preterm infants. However, since sicker infants typically start feeding later, this may have influenced our results. Further research is needed to clarify this relationship [54].

In our study, lower weight gain in the first 30 days of life was associated with the development and progression of ROP, although not independently. Slow postnatal weight gain has emerged as a potential predictor of ROP [55]. While low insulin-like growth factor-1 (IGF-1) levels, which are not routinely measured in NICUs, are associated with ROP risk, weight gain can serve as a proxy due to its correlation with IGF-1 and infant growth [56,57,58,59]. The WINROP model, introduced in 2006, uses postnatal weight gain to predict ROP risk and has been validated in various populations [60,61,62]. Our findings highlight the potential for refining ROP screening guidelines by incorporating weight gain data to reduce unnecessary examinations.

Dystocic birth was independently associated with type 1 ROP in our study. Of the 327 dystocic births, 322 (98.5%) were cesarean deliveries. The link between delivery type and ROP may be influenced by the maternal conditions leading to cesarean sections [63,64].

Resuscitation with endotracheal intubation at birth was significantly associated with ROP development and its progression, likely due to the associated risks of hypoxia and hyperoxia [65]. Low Apgar scores have been suggested as a potential risk factor for ROP development [66]; however, our study did not find an independent relationship. While bronchopulmonary dysplasia, which shares similar pathophysiological mechanisms with ROP in premature infants [67], was associated with ROP development and progression, this association was not independent.

In previous research, we identified an association between specific blood count parameters and ROP [20]. Our current study suggests that serum creatinine is associated with ROP development and progression. Serum urea, another potential ROP biomarker [68], was also associated with the development of ROP. Interestingly, elevated total bilirubin levels were associated with a lower risk of ROP, possibly due to its antioxidant effects or higher RBC counts [69]. Hyperglycemia in the first three weeks of life was associated with ROP development and progression, although a meta-analysis found otherwise [70]. None of these associations were independent.

The duration of hospitalization was significantly longer in infants with ROP, particularly those requiring treatment, reflecting greater morbidity.

Our study found that progression to type 1 ROP was associated with chronic arterial hypertension, lower maternal education levels, and Portuguese ancestry. Maternal chronic hypertension was independently associated with type 1 ROP, whereas pregnancy-induced hypertension appeared to have a protective effect, possibly due to preeclampsia’s role in stimulating HbF synthesis [71]. Although the impact of maternal education and socioeconomic status on ROP remains unclear, it may influence the quality of prenatal and postnatal care [72]. Additionally, since type 1 ROP is reported to be less common among Black preterm infants [73], the higher prevalence of type 1 ROP among those of Portuguese ancestry may be related to the greater proportion of African origin in the non-Portuguese population in Portugal.

Assisted reproductive techniques were independently associated with a reduced risk of ROP requiring treatment, likely due to close monitoring and less severe associated conditions [74,75].

Of the 337 eyes with ROP, 288 (85.5%) were in Zone II and 23 (6.8%) in Zone I. Among the 42 treated eyes, 10 (23.8%) had Zone I ROP and 32 (76.2%) had Zone II ROP. No ROP stage 4 or 5 cases were observed, and all treated cases achieved remission, underscoring the effectiveness of timely treatment and screening. Infants with type 1 ROP were diagnosed at stage 1 earlier than those who did not require treatment.

All cases treated with laser photocoagulation achieved remission, and while anti-VEGF therapy was also effective, four cases experienced recurrences that were successfully managed. These findings underscore the importance of close follow-ups to ensure long-term treatment success, particularly in patients treated with anti-VEGF agents, as recurrences can occur, with a significant number of these failures reported after 50 weeks postmenstrual age [76]. Current Portuguese guidelines recommend screening preterm infants with a GA < 32 weeks, BW < 1500 g, and BW < 2000 g with a prolonged need for supplemental oxygen or unstable clinical evolution [77]. Our study detected ROP in 2.2% of infants over 32 weeks GA, but none required treatment. Similarly, 1.3% of infants with BW > 1500 g developed ROP, with no cases requiring treatment. Our findings support the use of algorithmic models based on GA, BW, and weight gain to optimize ROP screening [78].

Efficient ROP screening should identify all at-risk preterm infants while minimizing the potential harms of over-screening, such as discomfort to the infant, excessive use of human resources, and high costs. Making an informed decision about the optimal screening method requires a thorough understanding and comprehensive analysis of the risk factors for ROP and its progression.

This study underscores the necessity of a multifaceted approach to ROP prevention, focusing on key risk factors such as oxygen management, transfusions, and sepsis prevention. Optimizing neonatal care practices, including perinatal interventions like delayed cord clamping and meticulous oxygen management, is essential in reducing ROP incidence by minimizing fluctuations in oxygen levels that contribute to oxidative stress in the retina. Additionally, strict infection control and careful management of RBC and platelet transfusions are crucial, as these directly affect retinal oxygenation and vascularization. These findings, consistent with previous research [13], highlight the need for ongoing refinement of neonatal care practices to prevent ROP progression and improve outcomes for preterm infants.

Future large-scale studies should validate these risk factors and explore innovative strategies, such as minimizing adult RBC transfusions, to enhance ROP management. This could lead to more effective screening models and tailored interventions, ultimately reducing the burden of ROP in preterm infants.

Integrating artificial intelligence (AI) into ROP screening and prevention offers transformative potential by improving early diagnosis and addressing the complex interplay of risk factors. AI can enhance screening accuracy by analyzing large NICU datasets and identifying patterns across diverse risk factors, such as transfusions and oxygen exposure, as well as by tracking the progression of ROP. This allows for more precise predictions and early identification of high-risk infants who could benefit from earlier or more frequent screenings. AI’s ability to automate retinal image analysis also ensures rapid and consistent assessments, reducing variability among examiners [79]. When combined with clinical data, AI can create comprehensive risk profiles for personalized care plans and monitor the effectiveness of interventions, dynamically adjusting treatment strategies to prevent ROP progression and optimize outcomes.

Our study has limitations. The multicentric design meant that different ophthalmologists conducted ROP screenings at various centers, which could introduce variability. Additionally, the exclusion of older preterm infants may have limited our ability to identify risk factors for ROP in this group. Despite these limitations, our prospective multicenter approach provides a representative sample from various regions of Portugal.

## 5. Conclusions

This study highlights several critical factors influencing the development and progression of ROP. Our findings suggest that a low GA and the number of RBC transfusions are potential predictors of both the onset and progression of ROP. Additionally, platelet transfusions, elevated maximum FiO_2_, and surfactant use were significantly associated with ROP development, while progression to severe ROP requiring treatment was associated with early and late sepsis, maternal chronic hypertension, and delayed initiation of enteral nutrition.

These results emphasize the importance of careful management in low-GA preterm infants, particularly concerning RBC and platelet transfusions, FiO_2_ levels, and vigilance for systemic inflammation. The association between postnatal weight gain and ROP reinforces the value of incorporating weight gain into predictive models, alongside GA and BW, to improve ROP screening accuracy. Moreover, the significant impact of GA and RBC transfusions underscores the need for strategies that preserve HbF percentages and optimize transfusion practices in preterm infants.

Integrating AI into ROP screening and prevention strategies offers significant potential for improving these efforts. AI-driven models can help develop personalized and effective screening protocols, improving early diagnosis and tailoring interventions to individual risk profiles. Future research should focus on validating these AI-driven approaches in diverse clinical settings and exploring their ability to transform neonatal care practices. Further research is necessary to validate these findings and refine predictive models. Enhancing screening accuracy and developing more effective intervention strategies are crucial to reducing the impact of ROP on preterm infants and improving their long-term visual outcomes.

## Figures and Tables

**Table 1:** Demographic and clinical characteristics of preterm infants according to the development (a) and progression (b) of retinopathy of prematurity (ROP).

	(a)				(b)			
Demographic and Clinical Characteristics	No ROP (*n* = 283) *n* (%) or Median (Q1–Q3)	ROP (*n* = 172) *n* (%) or Median (Q1–Q3)	*P*	*P* *	ROP Stages 1, 2, and 3 ^†^ (*n* = 151) *n* (%) or Median (Q1–Q3)	Type 1 ROP (*n* = 21) *n* (%) or Median (Q1–Q3)	*P*	*P* *
**BIRTH**								
**Eutocic birth**	76 (27.0%)	52 (30.2%)	0.454 ^§^	0.562	49 (32.5%)	3 (14.3%)	0.127 ^§^	**0.027**
**Gestational age (weeks)**	30.4 (29.1–31.5)	28.0 (26.4–29.3)	**<0.001** **^#^**	NA	28.3 (26.6–29.6)	26.1 (25.1–27.2)	**<0.001** **^#^**	**0.013**
≤28	58 (20.5%)	112 (65.1%)	**<0.001** **^§^**	NA	91 (60.3%)	21 (100%)	**0.002** **^§^**	NA
29–31	160 (56.5%)	50 (29.1%)			50 (33.1%)	0 (0%)		
≥32	65 (23.0%)	10 (5.8%)			10 (6.6%)	0 (0%)		
**Birth weight (g)**								
≤1000	54 (19.1%)	95 (55.2%)	**<0.001** **^§^**	0.055	76 (50.3%)	19 (90.5%)	**0.002** **^§^**	0.798
1001–1499	171 (60.4%)	71 (41.3%)			69 (45.7%)	2 (9.5%)		
≥1500	58 (20.5%)	6 (3.5%)			6 (4.0%)	0 (0%)		
**SGA**	81 (29.2%)	45 (26.9%)	0.603 ^§^	0.027	39 (26.5%)	6 (30.0%)	0.790 ^§^	0.128
**Gender**								
Female	136 (48.1%)	94 (54.7%)	0.177 ^§^	0.185	84 (55.6%)	10 (47.6%)	0.495 ^§^	0.487
Male	147 (51.9%)	78 (45.3%)			67 (44.4%)	11 (52.4%)		
**Resuscitation with endotracheal intubation**	39 (13.9%)	80 (46.8%)	**0.001** **^§^**	0.099	69 (46.0%)	11 (52.4%)	0.645 ^§^	**0.006**
**Oxygen**	191 (68.5%)	148 (86.5%)	**< 0.001** **^§^**	0.337	131 (87.3%)	17 (81.0%)	0.491 ^§^	0.120
**Maximum FiO_2_ (%)**	30.00 (27.25–40.00)	42.50 (30.00–67.50)	**<0.001** **^#^**	0.028	42.50 (30.00–72.50)	45.00 (30.00–52.50)	0.382 ^#^	0.064
**Apgar score 5th min < 7**	14 (4.9%)	26 (15.2%)	**<0.001** **^§^**	0.155	21 (14.0%)	5 (23.8%)	0.325 ^§^	0.699
**POSTNATAL**								
**Metabolic acidosis** (first 2 h of life)	2 (0.9%)	8 (5.7%)	**0.008** **^§^**	0.165	7 (5.6%)	1 (6.3%)	1.000 ^§^	0.752
Metabolic acidosis 1st day (between 2 and 24 h)	1 (0.6%)	3 (2.4%)	0.324 ^§^	0.098	3 (2.8%)	0 (0.0%)	1.000 ^§^	0.999
**Co-morbidities**								
Bronchopulmonary dysplasia moderate/ severe	28 (10.0%)	64 (37.4%)	**<0.001** **^§^**	0.741	47 (31.3%)	17 (81.0%)	**<0.001** **^§^**	0.053
Peri-intraventricular hemorrhage grade ≥ 2	20 (7.1%)	37 (21.8%)	**<0.001** **^§^**	0.384	31 (20.8%)	6 (28.6%)	0.407 ^§^	0.713
Cystic periventricular leukomalacia	4 (1.4%)	9 (5.3%)	**0.022** **^§^**	0.767	7 (4.7%)	2 (10.0%)	0.289 ^§^	0.993
Necrotizing enterocolitis	11 (3.9%)	15 (9.0%)	**0.035** **^§^**	0.831	14 (9.6%)	1 (5.0%)	1.000 ^§^	0.071
Early sepsis	26 (30.2%)	25 (22.5%)	0.252 ^§^	0.926	19 (20.7%)	6 (31.6%)	0.366 ^§^	**0.048**
Late sepsis	60 (69.8%)	86 (77.5%)	0.252 ^§^	0.926	73 (79.3%)	13 (68.4%)	0.366 ^§^	**0.048**
Hemodynamically significant patent ductus arteriosus	20 (7.1%)	43 (25.1%)	**<0.001** **^§^**	0.095	31 (20.7%)	12 (57.1%)	**0.001** **^§^**	0.829
Hyperbilirubinemia with phototherapy	237 (84.0%)	162 (95.9%)	**<0.001** **^§^**	0.275	141 (95.3%)	21 (100.0%)	0.598 ^§^	0.999
Number of days with hyperglycemia (in the first 21 days)	0.00 (0.00–2.00)	2.00 (0.00–5.00)	**<0.001** **^#^**	0.485	2.00 (0.00–4.00)	5.00 (3.00–7.00)	**0.001** **^#^**	0.565
**Treatments**								
RBC transfusions	50 (17.7%)	114 (66.3%)	**<0.001** **^§^**	NA	94 (62.3%)	20 (95.2%)	**0.002** **^§^**	NA
Platelet transfusions	9 (3.2%)	37 (21.5%)	**<0.001** **^§^**	0.008	29 (19.2%)	8 (38.1%)	0.084 ^§^	0.332
Days of invasive and non-invasive mechanical ventilation	3.00 (1.00–11.00)	32.00 (7.00–51.00)	**<0.001** **^#^**	0.217	26.00 (6.00–48.00)	54.00 (39.50–78.00)	**<0.001** **^#^**	0.481
Days of invasive mechanical ventilation	0.00 (0.00–1.00)	0.00 (3.00–17.75)	**<0.001** **^#^**	0.096	2.00 (0.00–15.00)	27.00 (12.50–40.50)	**<0.001** **^#^**	0.541
Surfactant	92 (33.6%)	127 (74.3%)	**<0.001** **^§^**	0.015	108 (72.0%)	19 (90.5%)	0.107 ^§^	0.793
Erythropoietin or darbepoetin	9 (3.2%)	13 (7.6%)	**0.043** **^§^**	0.959	11 (7.4%)	2 (9.5%)	0.665 ^§^	0.719
Systemic corticosteroid	14 (5.0%)	44 (25.7%)	**<0.001** **^§^**	0.891	31 (20.7%)	13 (61.9%)	**<0.001** **^§^**	0.133
Inhaled corticosteroid	15 (6.0%)	37 (25.2%)	**< 0.001** **^§^**	0.242	29 (22.3%)	8 (47.1%)	**0.038** **^§^**	0.239
Bronchodilator	16 (5.7%)	29 (16.9%)	**<0.001** **^§^**	0.595	24 (15.9%)	5 (23.8%)	0.358 ^§^	0.872
Non-steroidal anti-inflammatory	13 (4.6%)	32 (18.7%)	**<0.001** **^§^**	0.319	24 (16.0%)	8 (38.1%)	**0.031** **^§^**	0.944
Diuretics	50 (17.7%)	100 (58.1%)	**<0.001** **^§^**	0.157	83 (55.0%)	17 (81.0%)	**0.032** **^§^**	0.792
**Weight increase**								
Mean daily weight increase up to the 10th day	0.000 (−6.083–5.575)	−2.900 (−7.944–1.273)	**<0.001** **^#^**	0.072	−3.300 (−8.136–0.782)	−1.500 (−6.841–2.875)	0.325 ^¥^	0.089
Mean daily weight increase from the 11th to the 20th day	22.753 (16.861–30.000)	15.000 (10.417–21.700)	**<0.001** **^#^**	0.912	15.929 (12.000–22.000)	9.778 (6.075–14.750)	**0.002** **^¥^**	0.426
Mean daily weight increase from the 21st to the 30th day	28.700 (21.438–35.667)	20.909 (13.300–28.800)	**<0.001** **^#^**	0.913	21.214 (15.000–29.706)	12.000 (5.000–21.650)	**<0.001** **^¥^**	0.253
**Nutrition**								
Day of the start of trophic enteral nutrition	2.00 (2.00–3.00)	3.00 (2.50–6.00)	**<0.001** **^#^**	0.228	2.00 (2.00–3.00)	3.00 (2.50–6.00)	**<0.001** **^#^**	**0.032**
Day of the start of nutritious enteral nutrition	4.00 (3.00–6.00)	9.50 (5.25–19.25)	**<0.001** **^#^**	0.681	5.00 (4.00–7.00)	9.50 (5.25–19.25)	**<0.001** **^#^**	**0.022**
Day of the start of total enteral nutrition	10.00 (7.00–14.75)	26.00 (16.50–46.50)	**<0.001** **^#^**	0.126	13.00 (9.00–20.00)	26.00 (16.50–46.50)	**0.028** **^#^**	0.932
Breast milk	158 (82.7%)	123 (78.3%)	0.340 ^§^	0.745	110 (79.7%)	13 (68.4%)	0.251 ^§^	0.731
**Biochemical parameters**								
Urea (mg/dL)	49.0 (33.5–64.5)	61.6 (42.0–76.3)	**0.001** **^#^**	0.087	58.5 (41.0–74.8)	65.0 (49.7–93.7)	0.112 ^#^	0.956
Creatinine (mg/dL)	0.7 (0.6–0.8)	0.8 (0.6–0.9)	**<0.001** **^#^**	0.731	0.7 (0.6–0.8)	0.9 (0.8–1.0)	**0.005** **^#^**	0.092
Total bilirubin (mg/dL)	7.3 (5.8–8.7)	6.5 (5.3–7.5)	**0.001** **^#^**	0.825	6.6 (5.4–7.6)	5.6 (4.4–7.1)	0.087 ^#^	0.913
Direct bilirubin	0.5 (0.4–0.6)	0.7 (0.4–0.8)	**0.007** **^#^**	0.277	0.7 (0.5–0.8)	0.4 (0.4–0.8)	0.241 ^#^	0.095
Days of hospitalization	41.7 (29.4–53.2)	72.8 (53.2–94.5)	**<0.001** **^#^**	<0.001	68.6 (50.8–88.6)	104.0 (88.9–128.1)	**<0.001 ^#^**	0.082

N, number of individuals; NA, not applicable; *P*, *p*-value; *P* *, *p*-value adjusted for gestational age (GA) and number of red blood cell (RBC) transfusions. ^†^ Patients who do not meet the criteria for Type 1 ROP. ^§^ Chi-square test; ¥ Student’s *t*-test; ^#^ Mann–Whitney *U* test. *p*-values less than 0.05 are in bold.

**Table 2:** Clinical data related to supplemental oxygen therapy and the number of RBC transfusions to preterm infants according to the development (a) and progression (b) of ROP.

	(a)				(b)			
Clinical Data	No ROP (*n* = 283) Mean ± SD	ROP (*n* = 172) Mean ± SD	*P*	*P* *	ROP Stages 1, 2 and 3 ^†^ (*n* = 151) Mean ± SD	Type 1 ROP (*n* = 21) Mean ± SD	*P*	*P* *
Oxygen								
Maximum FiO_2_ 1st week	26.886 ± 43.155	31.345 ± 16.841	**<0.001** **^#^**	0.936	30.412 ± 17.027	38.347 ± 13.809	**0.001** **^#^**	0.431
Maximum FiO_2_ 2nd week	21.167 ± 5.110	28.512 ± 12.547	**<0.001** **^#^**	**0.045**	27.025 ± 10.478	39.364 ± 19.706	**0.001** **^#^**	0.132
Maximum FiO_2_ 3rd week	24.158 ± 6.103	34.464 ± 17.579	**<0.001** **^#^**	0.444	31.953 ± 16.014	46.537 ± 20.061	**<0.001** **^#^**	0.250
Blood transfusions								
Number of RBC transfusions	0.29 ± 0.835	2.19 ± 2.445	**<0.001** **^#^**	NA	1.80 ± 2.126	4.95 ± 2.837	**<0.001** **^#^**	NA
Number of platelet transfusions	0.05 ± 0.316	0.41 ± 1.025	**<0.001** **^#^**	**0.049**	0.34 ± 0.857	0.86 ± 1.797	**0.048** **^#^**	0.213
Number of plasma transfusions	0.01 ± 0.133	0.11 ± 0.452	**<0.001** **^#^**	0.929	0.11 ± 0.470	0.10 ± 0.301	0.748 ^#^	0.146

N, number of individuals; NA, not applicable; *P*, *p*-value; *P* *, *p*-value adjusted for GA and number of RBC transfusions; SD, standard deviation. ^†^ Patients who do not meet the criteria for Type 1 ROP. ^#^ Mann–Whitney *U* test. *p*-values less than 0.05 are in bold.

**Table 3:** Prenatal and maternal demographic and clinical characteristics according to the development (a) and progression (b) of ROP.

	(a)				(b)			
Demographic and Clinical Characteristics	No ROP (*n* = 283) *n* (%) or Median (Q1–Q3)	ROP (*n* = 172) *n* (%) or Median (Q1–Q3)	*P*	*P* *	ROP stages 1, 2, and 3 ^†^ (*n* = 151) *n* (%) or Median (Q1–Q3)	Type 1 ROP (*n* = 21) *n* (%) or Median (Q1–Q3)	*P*	*P* *
**Maternal age**								
<18 years	1 (0.4%)	2 (1.2%)	0.534 ^§^	0.598	2 (1.3%)	0 (0%)	0.749 ^§^	0.607
18–35 years	206 (72.8%)	121 (70.3%)			105 (69.5%)	16 (76.2%)		
>35 years	76 (26.9%)	49 (28.5%)			44 (29.1%)	5 (23.8%)		
**Non-Portuguese family ancestry**	66 (23.6%)	50 (29.4%)	0.183 ^§^	0.160	48 (32.2%)	2 (9.5%)	**0.039** **^§^**	**0.045**
**Level of education**								
Primary education	57 (21.0%)	40 (24.8%)	0.516 ^§^	0.234	33 (23.4%)	7 (35.0%)	NA	**0.034**
Lower secondary education	8 (3.0%)	9 (5.6%)			8 (5.7%)	1 (5.0%)		
Upper secondary education	86 (31.7%)	50 (31.1%)			40 (28.4%)	10 (50.0%)		
Post-secondary non-tertiary education	2 (0.7%)	1 (0.6%)			1 (0.7%)	0 (0.0%)		
Tertiary education (any stage)	118 (43.5%)	61 (37.9%)			59 (41.8%)	2 (10.0%)		
**Behavioral habits**								
Tobacco	44 (16.2%)	30 (18.2%)	0.601 ^§^	0.179	26 (18.1%)	4 (19.0%)	1.000 ^§^	0.492
Alcohol	6 (2.2%)	3 (1.8%)	1.000 ^§^	0.678	3 (2.1%)	0 (0.0%)	1.000 ^§^	0.999
Illicit drugs	2 (0.7%)	3 (1.9%)	0.368 ^§^	0.511	2 (1.4%)	1 (5.3%)	0.314 ^§^	0.139
**Obstetric history**								
Number of previous births								
0	176 (62.4%)	105 (61.0%)	0.958 ^§^	0.963	98 (64.9%)	7 (33.3%)	**0.021** **^§^**	0.442
1–3	98 (34.8%)	62 (36,0%)			49 (32.5%)	13 (61.9%)		
≥4	8 (2.8%)	5 (2.9%)			4 (2.6%)	1 (4.8%)		
**Pregnancy data**								
Assisted reproduction techniques	34 (14.6%)	23 (14.5%)	1.000 ^§^	0.843	22 (15.6%)	1 (5.6%)	0.475 ^§^	**0.013**
Multiple births	97 (34.3%)	39 (22.8%)	**0.011** **^§^**	0.164	35 (23.3%)	4 (19.0%)	0.787 ^§^	0.227
**Pathologies in pregnancy**								
Chronic arterial hypertension	18 (6.4%)	25 (14.6%)	**0.005** **^§^**	0.137	19 (12.6%)	6 (30.0%)	0.084 ^§^	**0.049**
Pregnancy-induced hypertension	105 (37.1%)	44 (25.6%)	**0.012** **^§^**	0.722	39 (25.8%)	5 (23.8%)	1.000 ^§^	0.261
Chronic hypertension with preeclampsia	12 (4.2%)	11 (6.4%)	0.312 ^§^	0.561	8 (5.3%)	3 (14.3%)	0.136 ^§^	0.168
Diabetes	39 (13.8%)	22 (12.8%)	0.799 ^§^	0.948	18 (12.0%)	4 (19.0%)	0.483 ^§^	0.336
Chorioamnionitis	29 (10.3%)	25 (14.7%)	0.179 ^§^	0.872	23 (15.4%)	2 (9.5%)	0.743 ^§^	0.573

N, number of individuals; NA, not applicable; *P*, *p*-value; *P* *, *p*-value adjusted for GA and number of RBC transfusions. ^†^ Patients who do not meet the criteria for Type 1 ROP. ^§^ Chi-square test. *p*-values less than 0.05 are in bold.

**Table 4:** The outcome of different stages of ROP.

	Eyes (%)	Outcome
ROP stage 1	172 (18.9%)	Spontaneous regression on follow-up
ROP stage 2	92 (10.0%)	2 eyes treated with anti-VEGF
ROP stage 3	66 (7.4%)	12 eyes treated with anti-VEGF 24 eyes treated with laser photocoagulation 3 eyes treated with anti-VEGF and laser 1 eye treated with anti-VEGF, laser photocoagulation, and surgery

## Data Availability

The corresponding author can provide the datasets analyzed in this study upon reasonable request due to ethical reasons.

## Supplementary Materials

The following supporting information can be downloaded at: https://www.mdpi.com/article/10.3390/children11101154/s1, Table S1. Demographic and clinical characteristics of preterm infants according to the development (a) and progression (b) of retinopathy of prematurity (ROP). Table S2. Clinical data related to supplemental oxygen therapy and the number of RBC transfusions to preterm infants according to the development (a) and progression (b) of ROP. Table S3. Prenatal and maternal demographic and clinical characteristics according to the development (a) and progression (b) of ROP.
