# Supplementary material for: Retinopathy of Prematurity in Eight Portuguese Neonatal Intensive Care Units: Incidence, Risk Factors, and Progression—A Prospective Multicenter Study
Source: Children (Basel). 2024 Sep 24;11(10):1154. doi: 10.3390/children11101154 (PMC11505647; doi:10.3390/children11101154)
Supplement: Supplementary file 1 [file children-11-01154-s001.zip › Table S1.docx]

|  | | | **(a)** | | | | | | | | **(b)** | | | | | | |
| --- | --- | --- | --- | --- | --- | --- | --- | --- | --- | --- | --- | --- | --- | --- | --- | --- | --- |
| **Demographic and clinical characteristics** | | | **No ROP (n=283)**  **n (%) or mediane (Q1-Q3)** | | **ROP (n=172)**  **n (%) or mediane (Q1-Q3)** | ***P*** | ***SE*** | **B OR** | **CI95% (LL – UL)** | ***P **** | **ROP stages 1, 2 and 3**^†^  **(n=151)**  **n (%) or mediane (Q1-Q3)** | **Type 1 ROP**  **(n=21)**  n (%) or mediane (Q1-Q3) | ***P*** | ***SE*** | **B OR** | **CI95% (LL – UL)** | ***P **** |
| **BIRTH** | | | | | | | | | | | | | | | | | |
| **Eutocic birth** | | | 76 (27.0%) | | 52 (30.2%) | 0.454**^§^** | 0.260 | -0.151 0.860 | (0.516-1.432) | 0.562 | 49 (32.5%) | 3 (14.3%) | 0.127**^§^** | 0.755 | 1.675 5.338 | (1.216-23.443) | **0.027** |
| **Gestational age (weeks)** | | | 30.4 (29.1-31.5) | | 28.0 (26.4-29.3) | **< 0.001^#^** | 0.399 |  |  | NA | 28.3 (26.6-29.6) | 26.1 (25.1-27.2) | **< 0.001^#^** | 0.196 | -0.488 0.614 | (0.418-0.901) | **0.013** |
| ≤28 | | | 58 (20.5%) | | 112 (65.1%) | **< 0.001^§^** |  | 0.494 1.639 | (0.749-3.585) | 0.216 | 91 (60.3%) | 21 (100%) | **0.002^§^** | 4481.739 | -17.587 NA | NA | NA |
| 29-31 | | | 160 (56.5%) | | 50 (29.1%) |  |  |  |  |  | 50 (33.1%) | 0 (0%) |  |  |  |  |  |
| ≥32 | | | 65 (23.0%) | | 10 (5.8%) |  |  |  |  |  | 10 (6.6%) | 0 (0%) |  |  |  |  |  |
| **Birth Weight (g)** ≤1000 | | | 54 (19.1%) | | 95 (55.2%) | **< 0.001^§^** | 0.226 | -0.195 0.822 | (0.528-1.282) | 0.055 | 76 (50.3%) | 19 (90.5%) | **0.002^§^** | 0.885 | -0.526 0.591 | (0.104-3.347) | 0.798 |
| 1001-1499 | | | 171 (60.4%) | | 71 (41.3%) |  |  |  |  |  | 69 (45.7%) | 2 (9.5%) |  |  |  |  |  |
| ≥1500 | | | 58 (20.5%) | | 6 (3.5%) |  |  |  |  |  | 6 (4.0%) | 0 (0%) |  |  |  |  |  |
| **SGA** | | | 81 (29.2%) | | 45 (26.9%) | 0.603**^§^** | 0.294 | 0.649 1.914 | (1.075-3.408) | **0.027** | 39 (26.5%) | 6 (30.0%) | 0.790**^§^** | 0.635 | 0.967 2.630 | (0.758-9.125) | 0.128 |
| **Gender** Female | | | 136 (48.1%) | | 94 (54.7%) | 0.177**^§^** | 0.238 | -0.315 0.730 | (0.458-1.163) | 0.185 | 84 (55.6%) | 10 (47.6%) | 0.495**^§^** | 0.536 | 0.372 1.451 | (0.508-4.149) | 0.487 |
| Male | | | 147 (51.9%) | | 78 (45.3%) |  |  |  |  |  | 67 (44.4%) | 11 (52.4%) |  |  |  |  |  |
| **Resuscitation with endotracheal intubation** | | | 39 (13.9%) | | 80 (46.8%) | **< 0.001^§^** | 0.291 | 0.480 1.615 | (0.913-2.856) | 0.099 | 69 (46.0%) | 11 (52.4%) | 0.645**^§^** | 0.699 | -1.911 0.148 | (0.038-0.582) | **0.006** |
| **Oxygen** | | | 191 (68.5%) | | 148 (86.5%) | **< 0.001^§^** | 0.307 | 0.295 1.344 | (0.736-2.454) | 0.337 | 131 (87.3%) | 17 (81.0%) | 0.491**^§^** | 0.840 | -1.307 0.271 | (0.052-1.403) | 0.120 |
| **Maximum FiO_2_ (%)** | | | 30.00 (27.25-40.00) | | 42.50 (30.00-67.50) | **< 0.001**^#^ | 0.005 | 0.012 1.012 | (1.001-1.022) | **0.028** | 42.50 (30.00-72.50) | 45.00 (30.00-52.50) | 0.382^#^ | 0.013 | -0.025 0.976 | (0.950-1.001) | 0.064 |
| **Apgar score 5^th^ min <7** | | | 14 (4.9%) | | 26 (15.2%) | **< 0.001^§^** | 0.423 | -0.601 0.548 | (0.240-1.255) | 0.155 | 21 (14.0%) | 5 (23.8%) | 0.325**^§^** | 0.711 | 0.275 1.317 | (0.327-5.302) | 0.699 |
|  |  |  | |  | | | | | | | | | | | | | |
| **Metabolic acidosis** (first 2 hours of life) | | | 2 (0.9%) | | 8 (5.7%) | **0.008^§^** | 0.860 | 1.193 3.299 | (0.611-17.809) | 0.165 | 7 (5.6%) | 1 (6.3%) | 1.000**^§^** | 1.160 | 0.366 1.442 | (0.149-14.000) | 0.752 |
| Metabolic acidosis 1^st^ day (between 2 and 24 hours) | | | 1 (0.6%) | | 3 (2.4%) | 0.324**^§^** | 1.213 | 2.004 7.420 | (0.689-79.967) | 0.098 | 3 (2.8%) | 0 (0.0%) | 1.000**^§^** | 22251.592 | -17.774 NA | NA | 0.999 |
| **Co-morbidities** | | | | | | | | | | | | | | | | | |
| Bronchopulmonary dysplasia moderate/ severe | | | 28 (10.0%) | | 64 (37.4%) | **< 0.001^§^** | 0.348 | -0.115 0.891 | (0.450-1.764) | 0.741 | 47 (31.3%) | 17 (81.0%) | **< 0.001^§^** | 0.656 | 1.269 3.556 | (0.982-12.873) | 0.053 |
| Peri-intraventricular hemorrhage grade ≥ 2 | | | 20 (7.1%) | | 37 (21.8%) | **< 0.001^§^** | 0.368 | 0.320 1.378 | (0.670-2.832) | 0.384 | 31 (20.8%) | 6 (28.6%) | 0.407**^§^** | 0.617 | -0.227 0.797 | (0.238-2.669) | 0.713 |
| Cystic periventricular leukomalacia | | | 4 (1.4%) | | 9 (5.3%) | **0.022^§^** | 0.790 | -0.233 0.792 | (0.168-3.721) | 0.767 | 7 (4.7%) | 2 (10.0%) | 0.289**^§^** | 0.953 | -0.009 0.991 | (0.153-6.420) | 0.993 |
| Necrotizing enterocolitis | | | 11 (3.9%) | | 15 (9.0%) | **0.035^§^** | 0.534 | -0.114 0.892 | (0.313-2.540) | 0.831 | 14 (9.6%) | 1 (5.0%) | 1.000**^§^** | 1.203 | -2.175 0.114 | (0.011-1.200 | 0.071 |
| Early sepsis | | | 26 (30.2%) | | 25 (22.5%) | 0.252**^§^** | 0.402 | -0.037 0.963 | (0.438-2.118) | 0.926 | 19 (20.7%) | 6 (31.6%) | 0.366**^§^** | 0.686 | 1.356 3.879 | (1.012-14.875) | **0.048** |
| Late sepsis | | | 60 (69.8%) | | 86 (77.5%) | 0.252**^§^** | 0.402 | 0.037 1.038 | (0.472-2.283) | 0.926 | 73 (79.3%) | 13 (68.4%) | 0.366**^§^** | 0.686 | -1.356 0.258 | (0.067-0.988) | **0.048** |
| Hemodynamically significant patent ductus arteriosus | | | 20 (7.1%) | | 43 (25.1%) | **< 0.001^§^** | 0.414 | -0.691 0.501 | (0.222-1.129) | 0.095 | 31 (20.7%) | 12 (57.1%) | **0.001^§^** | 0.607 | 0.131 1.140 | (0.347-3.748) | 0.829 |
| Hyperbilirubinemia with phototherapy | | | 237 (84.0%) | | 162 (95.9%) | **< 0.001^§^** | 0.477 | 0.521 1.684 | (0.661-4.289) | 0.275 | 141 (95.3%) | 21 (100.0%) | 0.598**^§^** | 14130.829 | 17.773 NA | NA | 0.999 |
| Number of days with hyperglycemia (in the first 21 days) | | | 0.00 (0.00-2.00) | | 2.00 (0.00-5.00) | **< 0.001^#^** | 0.046 | 0.032 1.032 | (0.944-1.129) | 0.485 | 2.00 (0.00-4.00) | 5.00 (3.00-7.00) | **0.001^#^** | 0.056 | -0.032 0.969 | (0.869-1.080) | 0.565 |

**Table S1.** Demographic and clinical characteristics of preterm infants according to the development (a) and progression (b) of retinopathy of prematurity (ROP).

**Table S1.** *Cont.*

|  | **(a)** | | | | | | | **(b)** | | | | | | |
| --- | --- | --- | --- | --- | --- | --- | --- | --- | --- | --- | --- | --- | --- | --- |
| **Demographic and clinical characteristics** | **No ROP (n=283)**  **n (%) or mediane (Q1-Q3)** | **ROP (n=172)**  **n (%) or mediane (Q1-Q3)** | ***P*** | ***SE*** | **B OR** | **CI95% (LL – UL)** | ***P **** | **ROP stages 1, 2 and 3**^†^  **(n=151)**  **n (%) or mediane (Q1-Q3)** | **Type 1 ROP**  **(n=21)**  n (%) or mediane (Q1-Q3) | ***P*** | ***SE*** | **B OR** | **CI95% (LL – UL)** | ***P **** |
| **Treatments** | | | | | | | | | | | | | | |
| RBC transfusions | 50 (17.7%) | 114 (66.3%) | **< 0.001^§^** |  |  |  | NA | 94 (62.3%) | 20 (95.2%) | **0.002^§^** |  |  |  | NA |
| Platelet Transfusions | 9 (3.2%) | 37 (21.5%) | **< 0.001^§^** | 0.490 | 1.292 3.641 | (1.394-9.513) | **0.008** | 29 (19.2%) | 8 (38.1%) | 0.084**^§^** | 0.583 | 0.566 1.761 | (0.562-5.523) | 0,332 |
| Days of invasive and non-invasive mechanical ventilation | 3.00 (1.00-11.00) | 32.00 (7.00-51.00) | **< 0.001^#^** | 0.008 | 0.010 1.010 | (0.994-1.027) | 0.217 | 26.00 (6.00-48.00) | 54.00 (39.50-78.00) | **< 0.001^#^** | 0.013 | 0.009 1.009 | (0.984-1.035) | 0.481 |
| Days of invasive mechanical ventilation | 0.00 (0.00-1.00) | 0.00 (3.00-17.75) | **< 0.001^#^** | 0.023 | 0.038 1.039 | (0.993-1.087) | 0.096 | 2.00 (0.00-15.00) | 27.00 (12.50-40.50) | **< 0.001^#^** | 0.020 | 0.012 1.013 | (0.973-1.054) | 0.541 |
| Surfactant | 92 (33.6%) | 127 (74.3%) | **< 0.001^§^** | 0.266 | 0.648 1.911 | (1.134-3.220) | **0.015** | 108 (72.0%) | 19 (90.5%) | 0.107**^§^** | 0.869 | -0.228 0.796 | (0.145-4.368) | 0.793 |
| Erythropoietin or darbepoetin | 9 (3.2%) | 13 (7.6%) | **0.043^§^** | 0.508 | 0.026 1.027 | (0.379-2.779) | 0.959 | 11 (7.4%) | 2 (9.5%) | 0.665**^§^** | 0.921 | 0.332 1.394 | (0.229-8.471) | 0.719 |
| Systemic Corticosteroid | 14 (5.0%) | 44 (25.7%) | **< 0.001^§^** | 0.422 | 0.058 1.060 | (0.463-2.424) | 0.891 | 31 (20.7%) | 13 (61.9%) | **< 0.001^§^** | 0.554 | 0.831 2.296 | (0.776-6.793) | 0.133 |
| Inhaled corticosteroid | 15 (6.0%) | 37 (25.2%) | **< 0.001^§^** | 0.393 | 0.460 1.584 | (0.733-3.423) | 0.242 | 29 (22.3%) | 8 (47.1%) | **0.038^§^** | 0.583 | 0.687 1.987 | (0.633-6.237) | 0.239 |
| Bronchodilator | 16 (5.7%) | 29 (16.9%) | **< 0.001^§^** | 0.422 | -0.224 0.799 | (0.350-1.826) | 0.595 | 24 (15.9%) | 5 (23.8%) | 0.358**^§^** | 0.647 | -0.104 0.901 | (0.254-3.202) | 0.872 |
| Non-steroidal anti-inflammatory | 13 (4.6%) | 32 (18.7%) | **< 0.001^§^** | 0.461 | -0.459 0.632 | (0.256-1.560) | 0.319 | 24 (16.0%) | 8 (38.1%) | **0.031^§^** | 0.588 | 0.042 1.042 | (0.329-3.302) | 0.944 |
| Diuretics | 50 (17.7%) | 100 (58.1%) | **< 0.001^§^** | 0.290 | 0.410 1.507 | (0.854-2.659) | 0.157 | 83 (55.0%) | 17 (81.0%) | **0.032^§^** | 0.679 | -0.179 0.836 | (0.221-3.162) | 0.792 |
| **Weight increase** | | | | | | | | | | | | | | |
| Mean daily weight increase up to the 10^th^ day | 0.000 (-6.083-5.575) | -2.900 (-7.944-1.273) | **< 0.001^#^** | 0.014 | -0.025 0.975 | (0.948-1.002) | 0.072 | -3.300 (-8.136-0.782) | -1.500 (-6.841-2.875) | 0.325^¥^ | 0.040 | 0.069 1.071 | (0.990-1.159) | 0.089 |
| Mean daily weight increase from the 11^th^ to the 20^th^ day | 22.753 (16.861-30.000) | 15.000 (10.417-21.700) | **< 0.001^#^** | 0.003 | 0.000 1.000 | (0.995-1.006) | 0.912 | 15.929 (12.000-22.000) | 9.778 (6.075-14.750) | **0.002**^¥^ | 0.045 | -0.036 0.965 | (0.884-1.054) | 0.426 |
| Mean daily weight increase from the 21^st^ to the 30^th^ day | 28.700 (21.438-35.667) | 20.909 (13.300-28.800) | **< 0.001^#^** | 0.009 | 0.001 1.001 | (0.983-1.019) | 0.913 | 21.214 (15.000-29.706) | 12.000 (5.000-21.650) | **<0.001**^¥^ | 0.033 | -0.038 0.963 | (0.901-1.028) | 0.253 |
| **Nutrition** | | | | | | | | | | | | | | |
| Day of the start of trophic enteral nutrition | 2.00 (2.00-3.00) | 3.00 (2.50-6.00) | **< 0.001^#^** | 0.071 | -0.086 0.917 | (0.797-1.055) | 0.228 | 2.00 (2.00-3.00) | 3.00 (2.50-6.00) | **< 0.001^#^** | 0.089 | 0.191 1.210 | (1.016-1.442) | **0.032** |
| Day of the start of nutritious enteral nutrition | 4.00 (3.00-6.00) | 9.50 (5.25-19.25) | **< 0.001^#^** | 0.038 | -0.016 0.985 | (0.914-1.060) | 0.681 | 5.00 (4.00-7.00) | 9.50 (5.25-19.25) | **< 0.001^#^** | 0.050 | 0.114 1.121 | (1.016-1.236) | **0.022** |
| Day of the start of total enteral nutrition | 10.00 (7.00-14.75) | 26.00 (16.50-46.50) | **< 0.001^#^** | 0.023 | 0.035 1.036 | (0.990-1.083) | 0.126 | 13.00 (9.00-20.00) | 26.00 (16.50-46.50) | **0.028^#^** | 0.054 | 0.005 1.005 | (0.904-1.116) | 0.932 |
| Breast milk | 158 (82.7%) | 123 (78.3%) | 0.340**^§^** | 0.340 | 0.111 1.117 | (0.573-2.176) | 0.745 | 110 (79.7%) | 13 (68.4%) | 0.251**^§^** | 0.645 | -0.222 0.801 | (0.226-2.835) | 0.731 |
| **Biochemical parameters** | | | | | | | | | | | | | | |
| Urea (mg/dL) | 49.0 (33.5-64.5) | 61.6 (42.0-76.3) | **0.001^#^** | 0.006 | 0.020 1.02 | (1.01-1.03) | 0.087 | 58.5 (41.0-74.8) | 65.0 (49.7-93.7) | 0.112**^#^** | 0.012 | 0.019 1.020 | (0.996-1.043) | 0.956 |
| Creatinine (mg/dL) | 0.7 (0.6-0.8) | 0.8 (0.6-0.9) | **< 0.001^#^** | 0.126 | -0.087 0.92 | (0.72-0.99) | 0.731 | 0.7 (0.6-0.8) | 0.9 (0.8-1.0) | **0.005^#^** | 1.401 | 3.016 50.22 | (3.23-78.89) | 0.092 |
| Total bilirubin (mg/dL) | 7.3 (5.8-8.7) | 6.5 (5.3-7.5) | **0.001^#^** | 0.061 | -0.119 0.061 | (0.79-0.99) | 0.825 | 6.6 (5.4-7.6) | 5.6 (4.4-7.1) | 0.087**^#^** | 0.176 | -0.300 0.741 | (0.524-1.047) | 0.913 |
| Direct bilirubin | 0.5 (0.4-0.6) | 0.7 (0.4-0.8) | **0.007^#^** | 0.730 | 2.047 7.75 | (1.85-32.42) | 0.277 | 0.7 (0.5-0.8) | 0.4 (0.4-0.8) | 0.241**^#^** | 1.816 | -2.187 0.112 | (0.003-3.946) | 0.095 |
| Days of hospitalization | 41.7 (29.4-53.2) | 72.8 (53.2-94.5) | **<0.001^#^** | 0.006 | 0.058 1.06 | (1.05-1.07) | **<0.001** | 68.6 (50.8-88.6) | 104.0 (88.9-128.1) | **<0.001^#^** | 0.012 | 0.048 1.05 | (1.03-1.07) | 0.082 |

B, Coefficient β; **CI95%**, Confidence Interval of 95%; **LL, lower limit;** N, number of individuals; NA, not applicable; OR*,* odds ratio*; P*, *p*-*value; P**, *p-value* adjusted for gestational age (GA) and number of red blood cell (RBC) transfusions; SE, standard error; UL, Upper limit. † Patients who do not meet the criteria for Type 1 ROP. § Chi-square test; ¥ Student's *t*-test; # Mann–Whitney *U*test. *P-values* less than 0.05 are in bold.
