# Supplementary material for: Retinopathy of Prematurity in Eight Portuguese Neonatal Intensive Care Units: Incidence, Risk Factors, and Progression—A Prospective Multicenter Study
Source: Children (Basel). 2024 Sep 24;11(10):1154. doi: 10.3390/children11101154 (PMC11505647; doi:10.3390/children11101154)
Supplement: Supplementary file 1 [file children-11-01154-s001.zip › Table S2.docx]

**Table S2.** Clinical data related to supplemental oxygen therapy and the number of RBC transfusions to preterm infants according to the development (a) and progression (b) of ROP.

|  | **(a)** | | | | | | | **(b)** | | | | | | |
| --- | --- | --- | --- | --- | --- | --- | --- | --- | --- | --- | --- | --- | --- | --- |
| **Clinical data** | **No ROP (n=283)**  **Mean ± SD** | **ROP (n=172)**  **Mean ± SD** | ***P*** | **SE** | **B OR** | ***CI95% (LL-UL)*** | ***P **** | **ROP stages 1, 2 and 3**^†^  **(n=151)**  **Mean ± SD** | **Type 1 ROP**  **(n=21)**  Mean ± SD | ***P*** | ***SE*** | **B OR** | **CI95% (LL – UL)** | ***P **** |
| **Oxygen** | | | | | | | | | | | | | | |
| Maximum FiO_2_ 1^st^ week | 26.886 ± 43.155 | 31.345 ± 16.841 | **< 0.001^#^** | 0.003 | 0.000 1.000 | (0.993-1.007) | 0.936 | 30.412 ± 17.027 | 38.347 ± 13.809 | **0.001^#^** | 0.014 | 0.011 1.011 | (0.984-1.039) | 0.431 |
| Maximum FiO_2_ 2^nd^ week | 21.167 ± 5.110 | 28.512 ± 12.547 | **< 0.001^#^** | 0.026 | 0.051 1.053 | (1.001-1.107) | **0.045** | 27.025 ± 10.478 | 39.364 ± 19.706 | **0.001^#^** | 0.018 | 0.027 1.028 | (0.992-1.065) | 0.132 |
| Maximum FiO_2_ 3^rd^ week | 24.158 ± 6.103 | 34.464 ± 17.579 | **< 0.001^#^** | 0.023 | 0.018 1.018 | (0.973-1.065) | 0.444 | 31.953 ± 16.014 | 46.537 ± 20.061 | **< 0.001^#^** | 0.014 | 0.016 1.016 | (0.989-1.045) | 0.250 |
| **Blood transfusions** | | | | | | | | | | | | | | |
| Number of RBC transfusions | 0.29 ± 0.835 | 2.19 ± 2.445 | **< 0.001^#^** |  |  |  | NA | 1.80 ± 2.126 | 4.95 ± 2.837 | **< 0.001^#^** |  |  |  | NA |
| Number of platelet transfusions | 0.05 ± 0.316 | 0.41 ± 1.025 | **< 0.001^#^** | 0.297 | 0.585 1.796 | (1.003-3.215) | **0.049** | 0.34 ± 0.857 | 0.86 ± 1.797 | **0.048^#^** | 0.181 | 0.226 1.253 | (0.879-1.788) | 0.213 |
| Number of plasma transfusions | 0.01 ± 0.133 | 0.11 ± 0.452 | **< 0.001^#^** | 0.597 | -0.053 0.948 | (0.294-3.055) | 0.929 | 0.11 ± 0.470 | 0.10 ± 0.301 | 0.748**^#^** | 0.694 | -1.009 0.365 | (0.094-1.421) | 0.146 |

B, Coefficient β; **CI95%**, Confidence Interval of 95%; **LL, lower limit;** N, number of individuals; NA, not applicable; OR*,* odds ratio*; P*, *p-value; P**, *p-value* adjusted for GA and number of RBC transfusions; SD, standard deviation; SE, standard error; **UL, upper limit**. † Patients who do not meet the criteria for Type 1 ROP. # Mann–Whitney *U*test. *P-values* less than 0.05 are in bold.
