# Supplementary material for: Retinopathy of Prematurity in Eight Portuguese Neonatal Intensive Care Units: Incidence, Risk Factors, and Progression—A Prospective Multicenter Study
Source: Children (Basel). 2024 Sep 24;11(10):1154. doi: 10.3390/children11101154 (PMC11505647; doi:10.3390/children11101154)
Supplement: Supplementary file 1 [file children-11-01154-s001.zip › Table S3.docx]

**Table S3.** Prenatal and maternal demographic and clinical characteristics according to the development (a) and progression (b) of ROP.

|  | **(a)** | | | | | | | **(b)** | | | | | | |
| --- | --- | --- | --- | --- | --- | --- | --- | --- | --- | --- | --- | --- | --- | --- |
| **Demographic and clinical characteristics** | **No ROP (n=283)**  **n (%) or mediane (Q1-Q3)** | **ROP (n=172)**  **n (%) or mediane (Q1-Q3)** | ***P*** | ***SE*** | ***B OR*** | ***CI95% (LL-UL)*** | ***P **** | **ROP stages 1, 2, and 3**^†^ **(n=151)**  **n (%) or mediane (Q1-Q3)** | **Type 1 ROP (n=21)**  **n (%) or mediane (Q1-Q3)** | ***P*** | ***SE*** | ***B OR*** | **CI95% (LL – UL)** | ***P **** |
| **Maternal age** < 18 years | 1 (0.4%) | 2 (1.2%) | 0.534**^§^** | 0.255 | 0.190 1.209 | (0.733-1.994) | 0.598 | 2 (1.3%) | 0 (0%) | 0.749**^§^** | 0.581 | 0.384 1.468 | (0.470-4.580) | 0.607 |
| 18-35 years | 206 (72.8%) | 121 (70.3%) |  |  |  |  |  | 105 (69.5%) | 16 (76.2%) |  |  |  |  |  |
| > 35 years | 76 (26.9%) | 49 (28.5%) |  |  |  |  |  | 44 (29.1%) | 5 (23.8%) |  |  |  |  |  |
| **Non-Portuguese family ancestry** | 66 (23.6%) | 50 (29.4%) | 0.183**^§^** | 0.268 | 0.376 1.457 | (0.861-2.463) | 0.160 | 48 (32.2%) | 2 (9.5%) | **0.039^§^** | 0.827 | -1.659 0.190 | (0.038-0.962) | **0.045** |
| **Level of education** | | | | | | | | | | | | | | |
| Primary education | 57 (21.0%) | 40 (24.8%) | 0.516**^§^** | 0.077 | -0.092 0.912 | (0.785-1.061) | 0.234 | 33 (23.4%) | 7 (35.0%) | NA | 0.196 | -0.416 0.660 | (0.449-0.969) | **0.034** |
| Lower secondary education | 8 (3.0%) | 9 (5.6%) |  |  |  |  |  | 8 (5.7%) | 1 (5.0%) |  |  |  |  |  |
| Upper secondary education | 86 (31.7%) | 50 (31.1%) |  |  |  |  |  | 40 (28.4%) | 10 (50.0%) |  |  |  |  |  |
| Post-secondary non-tertiary education | 2 (0.7%) | 1 (0.6%) |  |  |  |  |  | 1 (0.7%) | 0 (0.0%) |  |  |  |  |  |
| Tertiary education (any stage) | 118 (43.5%) | 61 (37.9%) |  |  |  |  |  | 59 (41.8%) | 2 (10.0%) |  |  |  |  |  |
| **Behavioral habits** | | | | | | | | | | | | | | |
| Tabaco | 44 (16.2%) | 30 (18.2%) | 0.601**^§^** | 0.308 | 0.414 1.513 | (0.827-2.769) | 0.179 | 26 (18.1%) | 4 (19.0%) | 1.000**^§^** | 0.712 | 0.490 1.632 | (0.404-6.590) | 0.492 |
| Alcohol | 6 (2.2%) | 3 (1.8%) | 1.000**^§^** | 0.816 | -0.339 0.713 | (0.144-3.524) | 0.678 | 3 (2.1%) | 0 (0.0%) | 1.000**^§^** | 23076.155 | -18.244 NA | NA | 0.999 |
| Illicit drugs | 2 (0.7%) | 3 (1.9%) | 0.368**^§^** | 1.133 | 0.745 2.107 | (0.229-19.418) | 0.511 | 2 (1.4%) | 1 (5.3%) | 0.314**^§^** | 1.323 | 1.957 7.079 | (0.530-94.601) | 0.139 |
| **Obstetric history** | | | | | | | | | | | | | | |
| Number of previous births 0 | 176 (62.4%) | 105 (61.0%) | 0.958**^§^** | 0.236 | -0.083 0.920 | (0.579-1.462) | 0.963 | 98 (64.9%) | 7 (33.3%) | **0.021^§^** | 0.501 | 0.508 1.663 | (0.623-4.441) | 0.442 |
| 1-3 | 98 (34.8%) | 62 (36,0%) |  |  |  |  |  | 49 (32.5%) | 13 (61.9%) |  |  |  |  |  |
| ≥4 | 8 (2.8%) | 5 (2.9%) |  |  |  |  |  | 4 (2.6%) | 1 (4.8%) |  |  |  |  |  |
| **Pregnancy data** | | | | | | | | | | | | | | |
| Assisted reproduction techniques | 34 (14.6%) | 23 (14.5%) | 1.000**^§^** | 0.372 | 0.074 1.076 | (0.519-2.231) | 0.843 | 22 (15.6%) | 1 (5.6%) | 0.475**^§^** | 1.446 | -3.607 0.027 | (0.002-0.462) | **0.013** |
| Multiple births | 97 (34.3%) | 39 (22.8%) | **0.011^§^** | 0.279 | -0.389 0.678 | (0.392-1.172) | 0.164 | 35 (23.3%) | 4 (19.0%) | 0.787**^§^** | 0.724 | -0.875 0.417 | (0.101-1.724) | 0.227 |
| **Pathologies in pregnancy** | | | | | | | | | | | | | | |
| Chronic arterial hypertension | 18 (6.4%) | 25 (14.6%) | **0.005^§^** | 0.395 | 0.587 1.798 | (0.829-3.898) | 0.137 | 19 (12.6%) | 6 (30.0%) | 0.084**^§^** | 0.629 | 1.238 3.450 | (1.005-11.842) | **0.049** |
| Pregnancy-induced hypertension | 105 (37.1%) | 44 (25.6%) | **0.012^§^** | 0.276 | 0.098 1.103 | (0.642-1.893) | 0.722 | 39 (25.8%) | 5 (23.8%) | 1.000**^§^** | 0.664 | 0.747 2.111 | (0.574-7.756) | 0.261 |
| Chronic hypertension with preeclampsia | 12 (4.2%) | 11 (6.4%) | 0.312**^§^** | 0.535 | 0.311 1.364 | (0.478-3.892) | 0.561 | 8 (5.3%) | 3 (14.3%) | 0.136**^§^** | 0.884 | 1.217 3.378 | (0.598-19.088) | 0.168 |
| Diabetes | 39 (13.8%) | 22 (12.8%) | 0.799**^§^** | 0.347 | 0.055 1.057 | (0.536-2.086) | 0.948 | 18 (12.0%) | 4 (19.0%) | 0.483**^§^** | 0.721 | 0.694 2.002 | (0.487-8.233) | 0.336 |
| Chorioamnionitis | 29 (10.3%) | 25 (14.7%) | 0.179**^§^** | 0.352 | -0.057 0.945 | (0.474-1.882) | 0.872 | 23 (15.4%) | 2 (9.5%) | 0.743**^§^** | 0.833 | -0.469 0.625 | (0.122-3.198) | 0.573 |

B, Coefficient β; **CI95%,** Confidence Interval of 95%; **LL, lower limit;** N, number of individuals; NA, not applicable; OR*,* odds ratio*; P*, p-*value; P**, *p-value* adjusted for GA and number of RBC transfusions; SE, standard error; UL, Upper limit. † Patients who do not meet the criteria for Type 1 ROP. § Chi-square test*. P-values* less than 0.05 are in bold.
